# Advancing cardiac diagnostics: high-accuracy arrhythmia classification with the EGOLF-net model

**DOI:** 10.3389/fphys.2025.1613812

**Published:** 2025-06-27

**Authors:** Deepika Tenepalli, T. M. Navamani

**Affiliations:** School of Computer Science and Engineering (SCOPE), Vellore Institute of Technology (VIT), Vellore, Tamil Nadu, India

**Keywords:** arrhythmia, optimization, ECG, LSTM, gray wolf optimization, heart disease

## Abstract

**Introduction:**

Arrhythmia, characterized by irregular heartbeats, can range from harmless to potentially life-threatening disturbances in heart rhythm. Effective detection and classification of arrhythmias are crucial for timely medical intervention and management.

**Methods:**

This research utilizes the MIT-BIH Arrhythmia Database, a well acknowledged benchmark dataset, to train and validate the proposed EGOLFNet model, Enhanced Gray Wolf Optimization with LSTM Fusion Network. This model integrates advanced optimization techniques with deep learning to enhance diagnostic accuracy and robustness in arrhythmia detection. The methodology includes preprocessing the ECG signals to normalize and filter out noise, followed by feature extraction using statistical methods and wavelet transforms. The distinctive aspect of EGOLF-Net involves using Enhanced Gray Wolf Optimization to select optimal features, which are then processed by LSTM layers to capture temporal dependencies in the ECG data effectively.

**Results and Discussion:**

The model achieved an accuracy of 99.61%, demonstrating the potential of EGOLF-Net as a highly reliable tool for classifying arrhythmias, significantly advancing the capabilities of cardiology diagnostic systems. Thus the proposed EGOLF-Net model was developed and validated for accurately identifying heart arrhythmias using electrocardiogram (ECG) data.

## 1 Introduction

Arrhythmias are conditions characterized by irregular heart rhythms, varying from benign to potentially life-threatening (Antzelevitch and Burashnikov, 2011; Çınar and Tuncer, 2021; Attia et al., 2019). These anomalies arise from electrical disturbances that disrupt the normal rhythm of the heart, potentially leading to severe health complications such as stroke, heart failure, or sudden cardiac arrest. Over time, advances in medical technology and understanding of cardiovascular diseases have highlighted the critical need for accurate detection and timely treatment of arrhythmias, underscoring their significance in public health (Ferreira and Zanesco, 2016; Organization, 2020; Mc Namara et al., 2019).

The study focuses on five primary types of arrhythmias: Atrial Fibrillation (AFib), Supraventricular Tachycardia (SVT), Bradycardia, Premature Ventricular Contractions (PVCs), and Atrial Flutter (Luo et al., 2017). Each type presents unique challenges in detection and management, largely due to their distinct manifestations and impacts on cardiac function (Saini et al., 2013). For example, AFib is known for the rapid and irregular beating of the atrial chambers, while Bradycardia is characterized by abnormally slow heart rates (Saini et al., 2015; Islam et al., 2022; Behl et al., 2020; Councils, 2015). Accurate classification of these conditions is crucial for implementing appropriate therapeutic strategies (Nurmaini et al., 2020; Tenepalli and Navamani, 2024) proposed the ERFEX framework using XGBoost, RFE, and SHAP, achieving 98.23% accuracy. However, it still faces challenges such as overfitting and limited generalizability to diverse datasets.

Kumar et al. (2022) developed a deep learning model for detecting heart arrhythmias using data from IoT-connected ECG devices. Based on a modified Grey Wolf Optimizer (GWO) algorithm, this model achieved 95% accuracy. However, it faces challenges with computational cost and requires more sophisticated feature extraction techniques for improved performance (Singh and Mahapatra, 2024) investigates the use of Recurrent Convolutional Neural Networks (RCNN) optimized by a GWO algorithm for classifying heart arrhythmias. This model achieved an accuracy of 98% on standard datasets, surpassing traditional machine learning methods. However, it presents challenges, including high computational demands, the need for large amounts of labeled data, the risk of overfitting, and potential difficulties in practical implementation (Sinnoor and Janardhan, 2024). Proposed an LSTM-based arrhythmia classification system with optimized hyperparameters using the Runge-Kutta (RUN) optimizer, achieving 99. 87% precision by combining the preprocessing of the ECG signal (Liu et al., 2024) developed a lightweight deep learning model to detect heart arrhythmias using 12-lead ECG data. This model, optimized through techniques such as compact convolutions and identifying critical accuracy segments, achieves high accuracy (95.532%) while requiring fewer parameters. However, oversimplifying the model may lead to losing important information, potentially impacting its performance.

In recent years, optimization algorithms and machine learning models have become pivotal in enhancing the diagnosis and classification of medical conditions, including arrhythmias (Kohli and Verma, 2011). These technologies leverage vast amounts of data to uncover patterns undetectable by human experts, offering significant improvements in diagnostic accuracy and efficiency (Brenyo and Aktas, 2014; Karpagachelvi et al., 2010; Hagiwara et al., 2018). Integrating optimization techniques helps to fine-tune the model parameters and select relevant features, which is crucial for building robust predictive models (Hammad et al., 2022).

Electrocardiogram (ECG), a straightforward, inconspicuous, harmless method that measures the heart’s electrical activity, is a frequently used test to identify arrhythmias (Kıymaç and Kaya, 2023). Manual ECG interpretation is challenging due to waveform variability and subjective human assessment, prompting the development of automated machine-learning-based Cardio Vascular Disease (CVD) systems. Midani et al. (2023) developed a hybrid Deep Neural Network (DNN) model that effectively identifies arrhythmias in ECG data. It combines recurrent and contextual DNNs, achieving high accuracy, but may have computational limitations for real-time applications in portable devices or systems with restricted processing power. Kıymaç and Kaya (2023) proposed a memory-enhanced artificial hummingbird algorithm to optimize deep learning models for classifying cardiac arrhythmias from ECG data. It is fully automated, efficient, and robust but requires significant computational resources and large datasets for training. The 1D Convolutional Neural Network (CNN) method developed by Ahmed et al. (2023) has performed well in extracting important characteristics from ECG data. However, it had a compromised sensitivity, resulting in a greater occurrence of false negatives, a crucial issue in medical diagnosis. Furthermore, the approaches that use traditional Convolutional Neural Networks (CNNs) for image analysis, as introduced by Liu et al. (2022), showed effectiveness in extracting features. However, they faced challenges in terms of sensitivity, which could result in omitting important positive information necessary for clinical decision-making. Ojha et al. (2022) proposed a novel approach that combines autoencoders and SVM for feature extraction and classification. This method has demonstrated remarkable accuracy. However, it necessitates significant processing resources and meticulous tuning to prevent overfitting, particularly when dealing with smaller or imbalanced datasets.

The diverse range of approaches employed for arrhythmia detection, each with its advantages and limitations. While ongoing advancements in these methods show promise for improving heart health diagnoses, challenges such as computational requirements, mathematical complexity, and applicability to various datasets remain significant obstacles. Previous studies often encounter challenges such as limited accuracy due to inefficient feature optimization and the inability to capture ECG signal complexities fully. These constraints underscore the need for more robust and efficient models, creating opportunities for innovative solutions like the proposed EGOLF-Net model. This model aims to address these deficiencies by combining advanced optimization techniques with deep learning. The unique methodologies emphasized, such as improvements achieved through meta-heuristic algorithms and attention-based models, demonstrate the ongoing development in this field, emphasizing the need for further research and refinement to optimize patient care and clinical outcomes.

The main contributions of this research work are as follows:

• Proposed a novel fusion EGOLF-Net model that combines Enhanced Grey Wolf Optimization (EGOLF) with Long Short-Term Memory (LSTM) networks to predict arrhythmia.

• The sophisticated EGOLF-Net technique improves feature selection, diagnostic precision, and resilience in classifying arrhythmias.

• A thorough comparison analysis is done by evaluating the proposed model against existing research work in the literature.

• Conducted statistical significance analysis to verify the efficacy and reliability of the EGOLF-Net model for identifying arrhythmias.

The structure of this paper is organized as follows. The Related Works section discusses the existing methodologies in arrhythmia detection. The methodology section discusses the proposed methodology, introducing the EGOLF-Net model and describing its unique components and operational framework. The Result Analysis section presents the results of applying the EGOLF-Net model on ECG. Finally, the Conclusion and Future Scope are discussed.

## 2 Related works

In recent years, advancements in deep learning have significantly improved arrhythmia diagnosis. Researchers have explored various methods combining neural networks with advanced data processing to enhance accuracy. This section provides an overview of these methods, highlighting their strategies and performance in ECG data analysis.

Kumar et al. (2023) explored combining deep learning with fuzzy clustering to improve medical image diagnosis. Fuzzy clustering helped handle uncertainties in medical data, enhancing the model’s performance. However, using fuzzy logic can make the model less understandable due to complex rules and membership functions. To analyze Electrocardiogram (ECG) signals, Daydulo et al. (2023) used a time-frequency approach with deep learning models (ResNet 50 and AlexNet). Their approach achieved 99.2% overall accuracy, with high sensitivity and specificity. This method effectively captured the temporal and frequency aspects of ECG signals for accurate heart disease diagnosis. However, combining multiple deep learning models can make the model more computationally demanding, limiting its applicability in resource-constrained settings.

Tripathi et al. (2022) proposed a deep learning-based method for classifying cardiac arrhythmias using ECG signals, achieving high accuracy with reduced hardware needs. However, it requires large datasets and prolonged training. Soman and Sarath (2024) proposed a Chameleon-Sparrow Search Algorithm-based Deep Convolutional Neural Network (CsSA-based Deep CNN) for effectively classifying arrhythmia in ECG signals using wavelet-based preprocessing and feature extraction. However, further improvements are necessary to enhance its accuracy and address computational complexity concerns, particularly for real-time clinical applications. Cai et al. (2020) proposed Multi-ECGNet, a deep learning-based approach for multi-label ECG classification, effectively identifying multiple heart diseases simultaneously with high accuracy, surpassing human experts. However, its computational complexity and potential for misclassification may limit its real-world application. Ojha et al. (2022) extracted and classified features using an autoencoder and a Support Vector Machine (SVM). This combination made effective reduction of dimensionality and reliable classification possible. Autoencoder training can be computationally demanding, though, and it might need fine-tuning to prevent overfitting especially when working with smaller or imbalanced datasets. Haq et al. (2023) proposed the Reseek-Arrhythmia model. It effectively detects and classifies heart arrhythmias with high accuracy, but it may struggle with handling noisy data or distinguishing subtle arrhythmia patterns.

Mandala et al. (2024) proposed a new ensemble learning approach for arrhythmia detection using multi-lead ECG data, which achieves high sensitivity, specificity, and accuracy for detecting various arrhythmia classes. Jha (2024) provided a comprehensive review of existing machine-learning and deep-learning-based techniques for arrhythmia detection using ECG signals, highlighting their strengths, limitations, and potential areas for improvement. Begum and Singh (2024) proposed a CNN-based continual normalization classifier for arrhythmia detection, achieving 99.2% accuracy, outperforming existing methods. Glaser et al. (2024) systematically reviewed ML algorithms for predicting and detecting New-Onset Atrial Fibrillation (NOAF) in ICU patients, finding CatBoost and SVM as promising methods and highlighting their potential to improve clinical decision-making. Sposato et al. (2024) presented the management of atrial fibrillation (AF) in ischemic stroke/TIA patients, detailing the risk assessment and monitoring strategies based on different AF detection methods.

Yu et al. (2024) provided a comprehensive review of the pathophysiology and etiology of arrhythmia in COVID-19 patients, offering valuable insights for understanding the disease. Still, it may not detail the specific mechanisms underlying arrhythmia development. An automatic CNN arrhythmia classifier improved by a memory-enhanced artificial hummingbird algorithm was proposed by Kıymaç and Kaya (2023). Their methodology utilizes sophisticated algorithms to enhance the accuracy of classification. Nevertheless, the efficacy of the study may be limited by the dependence on memory-enhancement methods, which may not exhibit strong generalization capabilities across various datasets. Admass and Bogale (2024) Proposed a metaheuristic enhancement, combining optimal weighted feature integration with an attention-based hybrid deep learning model to categorize ECG signals. The objective of this approach is to improve the accuracy of categorization by combining different features and attention methodologies. Nevertheless, the intricacy of the model may result in higher computational requirements and possible constraints in handling various ECG signal specifications.

The existing literature review comprehensively explores contemporary methods for arrhythmia detection, focusing on various techniques and their effectiveness in utilizing electrocardiogram (ECG) data. Recurrent Deep Neural Networks (DNN), convolutional models like ResNet 50 and AlexNet Daydulo et al. (2023), and autoencoders integrated with Support Vector Machines (SVM) Ojha et al. (2022) have been widely adopted to enhance arrhythmia identification accuracy. While these approaches demonstrate excellent diagnostic performance, they often face computational complexity, interpretability issues, and resource requirements. For instance, the hybrid DNN model proposed by Midani et al. (2023) although achieving remarkable precision through temporal dynamics and contextual learning, is constrained by its high computational demands, limiting its suitability for real-time applications on portable devices. Similarly, Kumar et al. (2023) proposed a model that integrates deep learning with fuzzy clustering, improving diagnostic accuracy, but introduced interpretability challenges due to the complex nature of fuzzy logic rules and membership functions.

Recent artificial intelligence (AI) breakthroughs have markedly enhanced electrocardiogram (ECG)-based cardiac diagnostics, especially in arrhythmia diagnosis. Multiple studies have used machine learning and deep learning algorithms to automate ECG interpretation, exhibiting elevated accuracy levels. Muzammil et al. (2024) examined the efficacy of AI-enhanced ECG analysis in clinical diagnoses, highlighting that deep learning, especially convolutional neural networks (CNNs), may surpass conventional interpretation techniques by identifying patterns undetectable by human observers. Nevertheless, they emphasized significant limitations like dataset bias and interpretability challenges. Telmoud et al. (2024) assessed traditional machine learning models, including Decision Trees, Support Vector Machines, and Random Forests, to categorize ECG data. Their trials with the MIT-BIH dataset achieved accuracies ranging from 97% to 99%, especially with ensemble models. Nonetheless, these approaches often rely on manually crafted features and frequently fail to capture the temporal dynamics of ECG data, essential for detecting arrhythmic patterns.

Notwithstanding the substantial advancements in ECG-based arrhythmia classification, two significant deficiencies persist: the absence of cohesive optimization for feature selection and temporal modeling. Current models often depend on comprehensive feature sets lacking optimum selection or disregard temporal dynamics by concentrating on static ECG segments or images—insufficient resilience and adaptability. Numerous models are tailored to particular datasets, have inadequate noise filtering or generalization abilities, and often do not sustain high accuracy when used in clinical environments. Hence, we proposed EGOLF-Net (Enhanced Gray Wolf Optimization with LSTM Fusion Network), which addresses these gaps by introducing a hybrid framework that integrates Enhanced Gray Wolf Optimization (EGWO) for optimal feature selection, effectively reducing noise and redundancy in high-dimensional ECG data. LSTM layers to capture sequential dependencies inherent in ECG signals, enabling robust temporal pattern recognition, a fully integrated pipeline combining preprocessing, feature engineering, and learning to boost generalizability and diagnostic reliability.

## 3 Methodology

**FIGURE 1:**
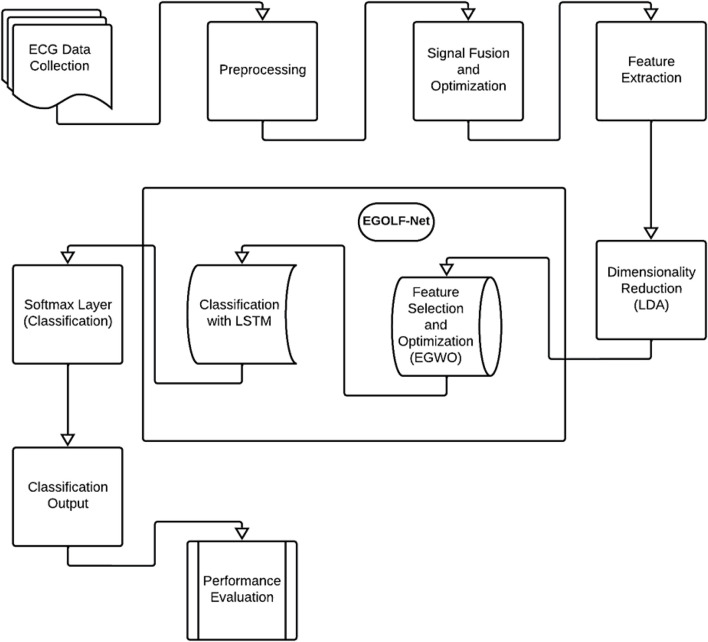
Proposed Block diagram.

The methodology for detecting and classifying arrhythmias using Electrocardiogram (ECG) data begins with collecting ECG signals from individuals showcasing a variety of cardiac conditions, encompassing both normal and abnormal rhythms as shown in Figure 1. These signals undergo preprocessing steps to refine data quality, including noise filtering, signal amplitude normalization, and segmentation to isolate specific features within the ECG trace. The signal fusion and optimization phase in the EGOLF-Net system combines multiple segments or different aspects of the ECG signals into a comprehensive signal. The optimized signal undergoes detailed feature extraction, involving statistical measures and frequency domain analysis, to capture the essential characteristics of the ECG data for accurate classification. Linear Discriminant Analysis (LDA) is applied for dimensionality reduction, emphasizing the most informative features for subsequent classification. This technique helps reduce the model’s computational complexity while preserving its discriminative power. Features extracted from the ECG signals serve as inputs to the proposed model EGOLF-Net model. After following the mechanism of EGWO, the selected features are fed to LSTM. The processed data is fed through LSTM layers, which learn complex patterns in the ECG signals. Finally, a SoftMax layer classifies the signals into different Arrhythmia types based on the learned patterns. The EGOLF-Net architecture effectively combines signal fusion, deep learning, and optimization techniques to enhance classification accuracy and overcome challenges associated with ECG data analysis.

### 3.1 Dataset description

Initially, ECG signals are collected from individuals with various cardiac conditions from the MIT-BIH dataset (Moody and Mark, 1992). A fundamental dataset in cardiac arrhythmia research, the MIT-BIH Arrhythmia Database provides digital recordings of ECG signals obtained from patients with different cardiac abnormalities. 48 half-hour sections of two-channel ambulatory ECG recordings make up the dataset, which cardiologists painstakingly annotate to identify specific arrhythmic patterns at a frequency of 360 Hz. Its comprehensive collection of 109,446 labeled beats includes atrial and ventricular premature beats, atrial fibrillation, ventricular fibrillation, and normal sinus rhythm. Beat classification and arrhythmia detection are supervised learning tasks made easier by carefully labeling every beat in this extensive repository. Using a subset of 3000 ECG signals from this repository, we set aside 70 for training, 15 for testing, and another 15 for validation. Such a prudent application of this dataset helps to advance the knowledge and diagnosis of cardiac arrhythmias in addition to benchmarking algorithms. The present implementation entails extracting 3,000 ECG segments from diverse patient records in the MIT-BIH Arrhythmia Database. The signals undergo processing using fusion and optimization methods to provide discriminative features, which are then used for model training and assessment. A randomized 70–15–15 partition (training, validation, testing) is executed on the whole dataset via randperm (), guaranteeing an equitable distribution of classes across the subsets.

The dataset division occurs at the sample level instead of the patient level, posing a possible danger of data leaking; signals from the same patient may be included in both the training and testing sets. This method is computationally efficient but may overestimate the model’s generalization capacity in practical situations. Future editions of the EGOLF-Net framework will provide patient-level stratified splitting, guaranteeing no overlap of patient data across the various groups. This improvement will bolster the clinical dependability and consistency of the model’s performance.

### 3.2 Feature extraction and selection

After data collection, preprocessing is performed to refine data quality. This includes normalization, noise filtering, and segmentation to isolate relevant features. Feature extraction techniques such as statistical measures and wavelet transforms are then applied, with dimensionality reduction techniques like Linear Discriminant Analysis (LDA) employed to emphasize informative features. The method’s core lies in a classification model called EGOLF-Net, where features are fed into a fusion network combining Enhanced Gray Wolf Optimization (EGWO) with Long Short-Term Memory (LSTM) architecture. Normalization is often applied to ensure that ECG signal amplitude varies within a specific range, generally between −1 and 1. It can be mathematically represented as in Equation 1:

$$x_{\mathrm{norm}}=\frac{\left(x-\min\left(x\right)\right)}{\left(\max\left(x\right)-\min\left(x\right)\right)}$$ (1)

where x is the original ECG signal, and $x_{\mathrm{norm}}$ is the normalized signal.

A common approach for filtering ECG signals is using a bandpass filter which might be represented by Equation 2

$$Y\left(f\right)=H\left(f\right)X\left(f\right)$$ (2)

where X(f) and Y(f) – Fourier transforms and H(f) - the frequency response.

After preprocessing, a signal fusion and optimization phase combine multiple segments or different aspects of the ECG signals into a comprehensive signal. This step enhances key features relevant to specific arrhythmias, ensuring focused analyses. The optimized signal then undergoes detailed feature extraction, involving statistical measures and frequency domain analysis via wavelet transforms. The energy of a signal segment can be calculated using Equation 3:

$$E=\Sigma_{n=1}^{N}|x\left[n\right]{|}^{2}$$ (3)

where x [n] represents the signal amplitude at sample n and N is the total number of samples in the segment.

Wavelet transform provides a way to analyze the signal at different scales and is defined as in Equation 4:

$$W_{x}\left(a,b\right)=\frac{1}{\sqrt{\left|a\right|}}\int_{-\infty }^{\infty }x\left(t\right)\varphi \left(\frac{t-b}{a}\right)dt\;$$ (4)

where a and b are the scale and translation parameters, φ(t) is the mother wavelet, and x(t) is the signal.

Features like mean and variance are computed as in Equations 5, 6:

Mean

$$\mu =\frac{1}{N}\overset{N}{\underset{n=1}{\sum }}x\left[n\right]$$ (5)

Variance

$$\sigma =\frac{1}{N}\overset{N}{\underset{n=1}{\sum }}{\left(x\left[n\right]-\mu \right)}^{2}$$ (6)

Linear Discriminant Analysis (LDA) is applied for dimensionality reduction, emphasizing the most informative features for subsequent classification. Texture analysis using Gray-Level Co-occurrence Matrix (GLCM) provides insights into ECG pattern complexity. GLCM, or Gray-Level Co-occurrence Matrix, is a technique used in image processing to analyze the spatial relationships of pixels based on their intensity values. Essentially, it quantifies how often different combinations of pixel intensities occur within an image. Texture features from the GLCM include Equations 7–10:

$$contrast=\overset{\mathrm{levels-1}}{\underset{i,j=0}{\sum }}P\left(i,j\right){\left(i-j\right)}^{2}$$ (7)

$$Energy=sum_{i}sum_{j}P{\left(i,j\right)}^{2}$$ (8)

$$Homogeneity=\frac{sum_{i}sum_{j}P\left(i,j\right)}{\left(1+{\left(i-j\right)}^{2}\right)}$$ (9)

$$correlation=\frac{sum_{i}sum_{j}\left(ij*P\left(i,j\right)-\mu_{i}\mu_{j}\right)}{\left(\sigma_{i}*\sigma_{j}\right)}$$ (10)

where.

μ : Mean, a measure of central tendency indicating the average value of the dataset.

σ : Variance, a measure of the spread or dispersion of the dataset around the mean.
P (i,j) :Probability matrix representing the occurrence of pixel intensity pairs in the image.
levels : Number of intensity levels in the image.
i and j : Intensity levels in the image.

In addition to the above features, the maximum, minimum, mean, median, and Root-Mean-Square (RMS) values are also extracted, which are active features.

The Enhanced Gray Wolf Optimization (EGWO) method is an improved version of the original GWO designed only for quickly selecting binary features. In EGWO, each wolf is a binary vector, and each bit shows whether or not a particular characteristic is chosen (1) or not (0). The technique starts with a controlled initialization, in which wolves are given random binary vectors that determine certain features (for example, 30) to ensure variety. A dynamic control parameter a goes down in a straight line across iterations, keeping the exploration and exploitation phases in balance. A backup plan deals with degenerate solutions: if a wolf does not choose any features, a valid random subset is returned to it to keep performance up. We use 5-fold cross-validation accuracy from a K-Nearest Neighbors (KNN) classifier to check how well each feature subset works. This helps us choose features that make predictions more accurate.

EGWO chooses the best features and then applies them to the training, validation, and test datasets. This makes the data much less complex. Using num2cell (), these filtered datasets are then changed into a sequence format that LSTM can use. The LSTM design starts with a sequenceInputLayer that matches the number of chosen features. Then, there is an LSTM layer with a set number of hidden units to collect patterns over time. To help with overfitting, a dropout layer is added. Then, for multi-class prediction, a fully connected layer, a softmax layer, and a classification layer are added. The Adam optimizer with L2 regularization is used to train the model, and a validation set is used to monitor how well it works. The final examination of the test data comprises checking the correctness of the classifications, visualizing the confusion matrix, and using multi-class ROC curves to see how well the model can tell the difference between classes. This combined EGWO-LSTM architecture provides a small, easy-to-understand, and high-performing pipeline that is perfect for biomedical classification tasks based on ECG.

### 3.3 EGOLF-NET model development

**FIGURE 2:**
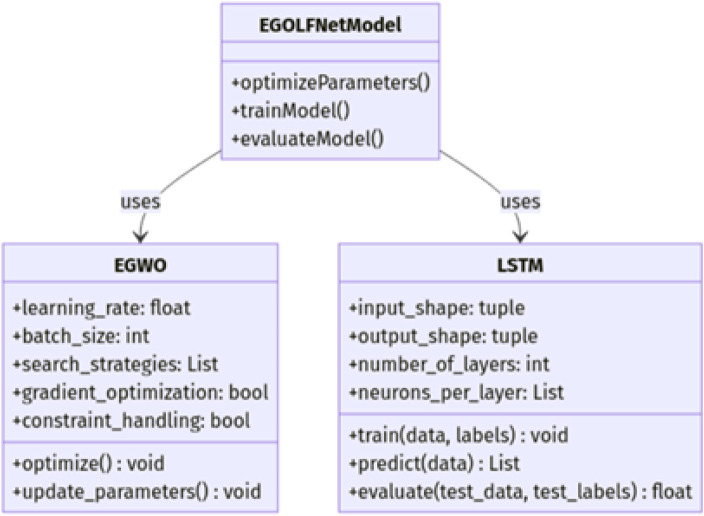
EGOLF-Net Model-class diagram..

The EGOLF-Net Model combines the Enhanced Gray Wolf Optimization (EGWO) algorithm with the Long Short-Term Memory (LSTM) model for effective classification tasks like ECG arrhythmia detection. Figure 2 shows the class diagram of the EGOLF-Net Model. This model is the main component that utilizes EGWO for optimizing key hyperparameters like learning rate and batch size through advanced search strategies, gradient-based optimization, and constraint handling. The LSTM class, responsible for sequence learning and classification, includes attributes such as input and output shapes, number of layers, and neurons per layer, with training, prediction, and evaluation methods. Figure 2 highlights how the EGOLF-Net Model uses both EGWO for optimization and LSTM for classification, demonstrating their roles and interactions within the architecture.

**FIGURE 3:**
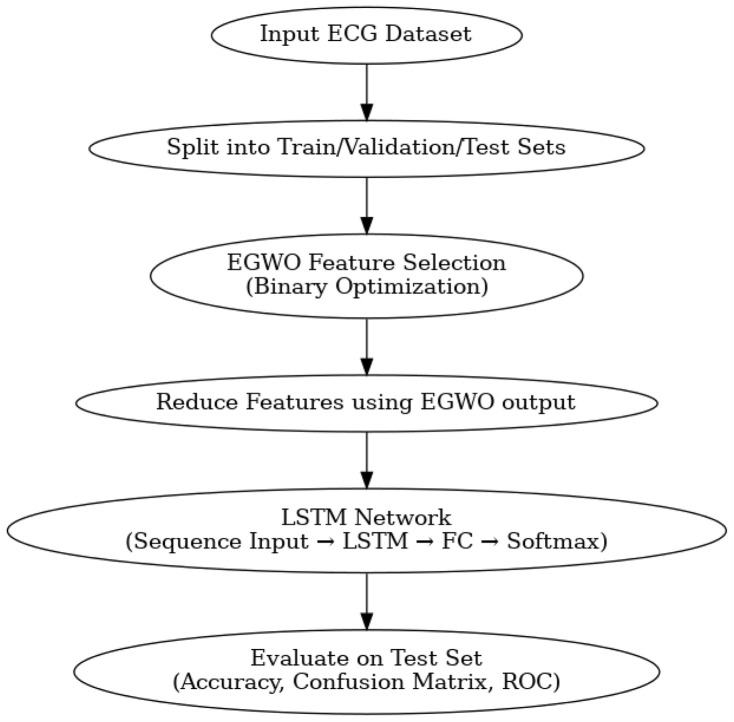
Flowchart of proposed work.

The choice of EGOLF-Net as the model for the arrhythmia classification network is apt for several reasons, as shown in Figure 3. Firstly, it succinctly encapsulates the core components of the methodology, combining Enhanced Gray Wolf Optimization (EGO) with LSTM Fusion, which is integral to the network’s effectiveness. Including ”O" for Optimization emphasizes the systematic refinement of the model’s performance, ensuring it leverages the most informative features for accurate classification. Additionally, the acronym is memorable and reflects the innovative approach taken in integrating advanced optimization techniques with deep learning methodologies.

The LSTM network consists of several critical layers, each playing a pivotal role in processing sequential data. The input layer is the initial point where the input sequence data is received. This layer prepares the data for further processing by the LSTM units. The core of the LSTM network lies in its LSTM layers, which consist of multiple LSTM units designed to capture temporal dependencies and learn patterns over time. Each LSTM unit includes a cell state and three gates: the forget gate, the input gate, and the output gate. The forget gate determines which information from the previous cell state should be discarded, while the input gate decides what new information should be added. The cell state is then updated accordingly. Finally, the output gate determines the next hidden state, crucial for making predictions.

Features extracted from the ECG signals serve as inputs to the proposed model EGOLF-Net model. After following the mechanism of EGWO, the selected features are fed to LSTM. The LSTM network processes extracted and selected feature sequences, learning to recognize patterns and features that indicate Arrhythmia or a healthy heart. It then classifies the type using the Equations 11–16.

LSTM Input Gate:

$$i_{t}=\sigma \left(W_{t}.\left[h_{t-1},x_{t}\right]+b_{i}\right)$$ (11)

LSTM Forget Gate:

$$f_{t}=\sigma \left(W_{f}.\left[h_{t-1},x_{t}\right]+b_{f}\right)$$ (12)

LSTM Cell State Update:

$$C_{t}^{\prime }=\tanh\left(W_{t}.\left[h_{t-1},x_{t}\right]+b_{c}\right)$$ (13)

$$C_{t}=f_{t}*C_{t-1}+I_{t}*C_{t}^{\prime }$$ (14)

LSTM Output Gate:

$$O_{t}=\sigma \left(W_{O}.\left[h_{t-1},x_{t}\right]+b_{O}\right)$$ (15)

$$h_{t}=O_{t}*\tanh\left(C_{t}\right)$$ (16)

Where.

• $W_{t},b_{i},b_{f},b_{c},b_{o}$ : Weights and biases used in the LSTM gates and cell state update equations.

• $h_{t-1},x_{t},i_{t},f_{t},C_{t},O_{t},h_{t}$ : variables representing the previous hidden state, current input, input gate, forget gate, cell state, output gate, and current hidden state of the LSTM network respectively.

After processing through the LSTM layers, the data reaches the output layer. The output layer provides the final prediction based on the learned patterns from the LSTM layers. It culminates in a SoftMax layer that classifies the signals based on Arrhythmia type. By combining EGWO for hyperparameter optimization and LSTM layers for capturing temporal dependencies, the EGOLF-Net Model offers a powerful and efficient framework for analyzing and classifying sequential data, such as ECG signals, to detect arrhythmias accurately. This integrated approach leverages optimization and deep learning techniques, resulting in a highly effective model for various applications. Equation 17 presents the mathematical expression for the softmax layer:

$$P\left(y_{k}|x\right)=\frac{\exp\left(O_{lk}\right)}{\sum_{n}\exp\left(O_{ln}\right)}$$ (17)

Where $P\left(y_{k}|x\right)$ :Probability of the output class given the input data.

This classification step is critical because it converts the complex, learned representations of ECG signals into a clinically relevant ailment related to Arrhythmia. Trained on labeled ECG records, this model identifies patterns associated with various arrhythmias and cardiac conditions. Upon classification, the system delivers detailed feedback, specifying the detected arrhythmia or cardiac condition. This feedback guides further clinical assessment and intervention, providing visual and textual information and annotations on ECG plots and alerts advising potential clinical actions based on classification results. Finally, performance evaluations are performed. This comprehensive methodology integrates advanced signal processing techniques and the proposed EGOLF-Net approach to provide a robust tool for diagnosing and understanding cardiac conditions through ECG data analysis, adaptable across various medical diagnostic platforms.

The novelty of the EGOLF-Net lies in its integration of Enhanced Gray Wolf Optimization with LSTM Fusion for arrhythmia classification. This hybrid approach combines the strengths of evolutionary optimization and deep learning, enabling the model to effectively learn complex patterns from ECG data while dynamically adapting its parameters for optimal performance. By leveraging the complementary advantages of both techniques, EGOLF-Net achieves enhanced accuracy and robustness in arrhythmia classification, paving the way for more reliable and efficient diagnostic tools in clinical practice. However, the model’s complexity can lead to increased computational requirements, which may pose challenges in resource-constrained environments. Additionally, while the EGWO enhances feature selection, it requires careful tuning to avoid potential overfitting, especially in datasets with high dimensionality or noise.

### 3.4 Algorithm of the proposed model

Algorithm 1 describes the application of the EGOLF-Net model, a complex machine-learning framework intended to improve the precision of arrhythmia detection with ECG data. The ECG data is loaded first, then pre-processed—normalization and band-pass filtering are two steps to get the data ready for feature extraction. The integration of Enhanced Gray Wolf Optimization (GWO), in which a population of simulated wolves iteratively optimizes the selection of features based on their fitness, finally identifying the most effective set for arrhythmia classification, makes this model unique. These optimized features are applied following the configuration of an LSTM network with multiple layers to handle temporal dependencies present in ECG data. The model’s accuracy, sensitivity, and specificity are assessed following training of the network with the Adam optimizer.

**1: Initialize EGOLF-Net Model Parameters**
Load ECG data: ECG_data← load (’ECG_dataset.mat’)
Set learning rate: learningRate←0.01

Set batch size: batchSize←100

Set regularization factor: regularizationFactor←0.001

**2: Preprocess ECG Data**
Normalize data: normalizedData← normalize (ECG_data)

Apply band-pass filter: filteredData← bandpassFilter ( normalizedData , [0.5,40] )
Segment data into heartbeats: segmentedData← segmentECG (filteredData)

**3: Feature Extraction**
Extract features: features← extractFeatures (segmentedData)

**4: Apply Enhanced GWO for Feature Optimization**
Set number of wolves: numWolves←30

Set maximum iterations: maxIter←100

Initialize alphawolf, betawolf, and deltawolf:
 
alphaWolf← initializeWolf (features)

 
betaWolf← initializeWolf (features)

 
deltaWolf← initializeWolf (features)

**For** iter=1 **to** maxIter **do**
** For** i=1 **to** numWolves **do**
  Update wolf position: wolf← updateWolfPosition (alphaWolf, betaWolf, deltaWolf, features )
   Evaluate fitness: fitness← evaluateFitness ( wolf , features )
   Update leaders: [alphaWolf, betaWolf, deltaWolf] ← updateLeaders ( wolf , fitness , alphaWolf, betaWolf, deltaWolf)
**  end for**
** end for**
Set optimal features: optimalFeatures←alphaWolf

**Step 5: Configure LSTM Layers**
Define LSTM layers: $layers=\left[\ldots \;\right.$ sequenceInputLayer (size (optimalFeatures,1)) lstmLayer (50,’OutputMode’,’last’) fullyConnectedLayer (5) five classes for arrhythmia types SoftmaxLayer classificationLayer];
Set training options: $options=trainingOptions{(}^{\prime }adam^{\prime },\ldots \;$ ’MaxEpochs’,30, ’MiniBatchSize’,batchSize, ’InitialLearnRate’,learningRate, L2Regularization’,regularizationFactor,’Plots’,’training-progress’);
**6: Train LSTM Network**
Train network: net← train network ( segmentedData , optimalFeatures , layers , options )

Algorithm 1 EGOLF-Net Model.

**7: Model Evaluation**
Classify data: predictedLabels← classify ( net , segmentedData )
 
performance← evaluateModel ( predictedLabels , ECG_data.Labels) Evaluate model performance
**8: Output Results**
disp(’Model training complete.’)
disp ([’Accuracy: ’, num2str (performance.accuracy) ])
disp ([’Sensitivity: ’, num2str (performance.sensitivity) ])
disp ([’Specificity: ’, num2str (performance.specificity) ])

The fitness function used in EGOLF-Net is the Accuracy Maximization Function which aims to maximize the classification accuracy of the Long Short-Term Memory (LSTM) model by optimizing feature subsets from the Electrocardiogram (ECG) signals. The search space, the High-Dimensional Feature Combination Space, includes all possible combinations of extracted features such as time-domain, frequency-domain, and non-linear features from the ECG signals, making it complex and vast. Simple search methods like Sequential Feature Selection (SFS) and Grid Search Cross-Validation are computationally expensive and impractical for navigating this complex space due to their exhaustive and non-adaptive nature. In contrast, Enhanced Gray Wolf Optimization (EGWO) efficiently navigates the High-Dimensional Feature Combination Space using heuristic-driven exploration and exploitation strategies, significantly reducing computation time while enhancing the LSTM model’s predictive performance. This approach ensures the selection of the most relevant feature subsets, thereby improving the overall accuracy and efficiency of the model.

## 4 Results and analysis

The experiments were conducted in a MATLAB R2023a environment using the system shown in Table 1. All ECG signal preprocessing, feature extraction, EGWO optimization, and LSTM training were performed on the same platform. To enhance the performance of the EGOLF-Net model in categorizing arrhythmias, the training parameters are carefully set as shown in Table 2. The learning rate is configured at 0.01, enabling the model to significantly adjust weights during each iteration while maintaining a balance between convergence speed and stability. To achieve a suitable level of learning without overfitting, the model is trained for 30 epochs with a batch size of 100, effectively balancing training speed and memory efficiency. An L2 regularization factor of 0.001 punishes big weights to improve the model’s generalization capabilities further. A total of 50 LSTM units are configured to ensure the network can accurately capture the temporal relationships in ECG data.

**TABLE 1:** Hardware specifications and training time.

Parameter	Specification
Processor	Intel Core i7 (12-core) @ ∼ 2.6 GHz
RAM	16 GB DDR4
Operating System	Windows 10, 64-bit
Software Environment	MATLAB R2023a
ECG Signals Used	3,000 (MIT-BIH Arrhythmia Database)
Feature Extraction Time	∼ 6 min
EGWO Optimization Time	∼ 8 min
LSTM Training Time	∼ 7 min (30 epochs)
Total Training Duration	∼ **21 min**

**TABLE 2:** Training parameters of EGOLF-Net model.

Parameter	Value
Learning Rate	0.01
Batch Size	100
Number of Epochs	30
L2 Regularization Factor	0.001
Number of LSTM Units	50
Initial Weights Initialization	He Normal
Optimizer	Adam
Dropout Rate	0.2
Feature Selection Method	Enhanced Gray Wolf Optimization (EGWO)

The normal initialization is employed for the weights, specifically designed for ReLU activation functions, to ensure a consistent signal variance across the network. To avoid overfitting, the Adam optimizer is selected for its adjustable learning rate, effective management of sparse gradients, and a dropout rate of 0.2. The feature selection process is guided by Enhanced Gray Wolf Optimization (EGWO), which iteratively improves the input features to guarantee that the most pertinent data is included in the classification procedure. By meticulously choosing these parameters, the EGOLF-Net model’s resilience and effectiveness in real-time arrhythmia detection are significantly improved.

The diagnostic system’s performance is assessed using accuracy, sensitivity, specificity, predictive values, and likelihood ratios, as defined in Equations 18–21, ensuring its reliability and efficacy for clinical application.

Accuracy

$$Accuracy=\frac{TP+TN}{TP+TN+FP+FN}$$ (18)

Sensitivity (True Positive Rate):

$$Sensitivity=\frac{TP}{TP+FN}$$ (19)

Specificity (True Negative Rate):

$$Specificity=\frac{TN}{TN+FP}$$ (20)

Area Under the ROC Curve (AUC):

$$AUC=\overset{1}{\underset{0}{\int }}TPR\left(d^{\prime }\right)dFPR$$ (21)

where TPR is depicted as the True Positive Rate whereas FPR is the False Positive Rate as a function of threshold t.

**FIGURE 4:**
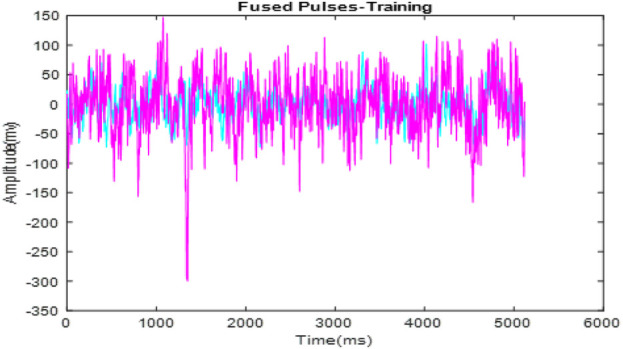
Fusion of signal pulses.

**FIGURE 5:**
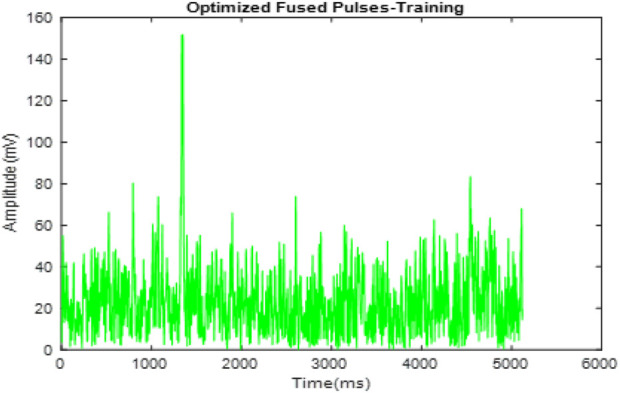
Optimization of fused pulses.

Figure 4 presents the fused ECG signals. This fusion process integrates signals from multiple subjects into a single, comprehensive dataset, shown here with overlaid signals that enhance the representation for analysis. Figure 5 shows the optimized fused signals from Figure 4, where optimization processes have been applied to enhance diagnostic features, reduce noise, and suppress irrelevant information. The result is a cleaner, more diagnostic-friendly signal, highlighting key features crucial for effective arrhythmia detection.

**FIGURE 6:**
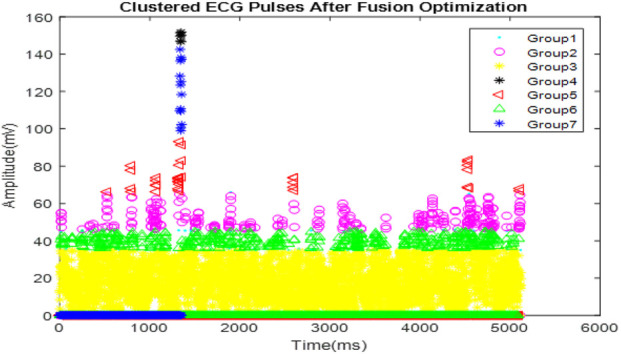
Clustered ECG signals after optimization.

Figure 6 illustrates the clustering of optimized ECG signals, with various symbols and colors denoting different clusters. These clusters are formed based on characteristics identified in the ECG signals that correspond to distinct arrhythmic conditions or signal patterns.

Table 3 lists the dynamic features extracted from the ECG signals of ten subjects (S-1 to S-10). It includes maximum and minimum values, mean, median, and RMS (Root Mean Square). These features capture the essential statistical properties of the ECG signals which are critical for training the EGOLF-Net in pattern recognition associated with normal and abnormal cardiac functions.

**TABLE 3:** Features extracted.

Dynamic features extracted	S-1	S-2	S-3	S-4	S-5	S-6	S-7	S-8	S-9	S-10
Max and Min	198.76	181.42	253.63	257.11	181.42	259.11	176.76	178.96	257.71	171.42
Mean	38.21	59.39	68.87	73.23	59.39	74.23	29.21	32.21	73.24	60.39
Median	32.90	53.68	64.42	63.02	53.68	65.05	27.90	29.84	65.95	53.68
RMS	46.40	66.95	79.00	85.37	66.95	84.34	38.40	37.50	83.54	67.90

**FIGURE 7:**
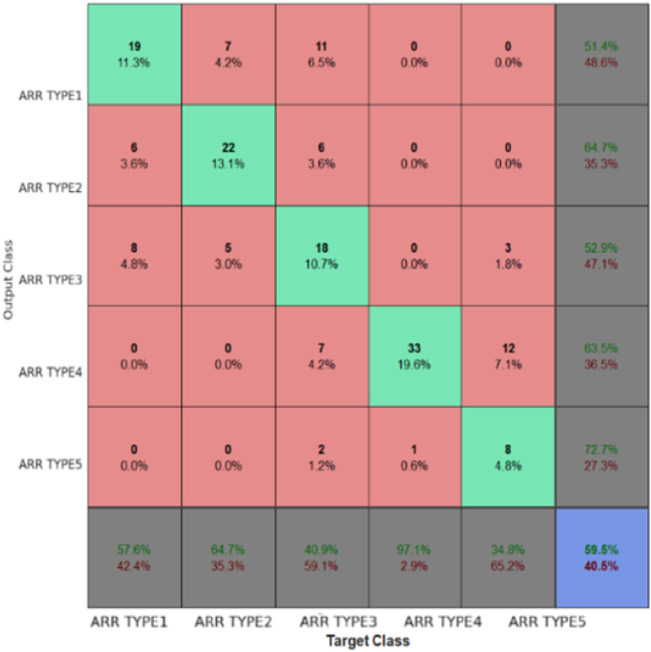
Confusion matrix for trained data.

**FIGURE 8:**
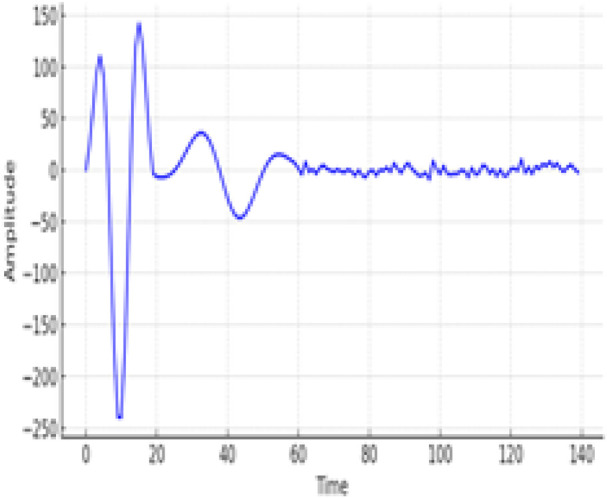
SVT type of Arrhythmia Detected for Subject two.

Figure 7 is a confusion matrix that categorizes the classification performance of the EGOLF-Net model across five types of arrhythmias. It quantifies the model’s accuracy in predicting each arrhythmia type, with true positives highlighted on the diagonal and classification errors in the off-diagonal cells. Figure 8 specifically demonstrates a Supraventricular Tachycardia (SVT) event detected in the ECG data for Subject 2. It highlights how the model isolates and identifies specific types of arrhythmias based on the learned patterns from the training data.

**FIGURE 9:**
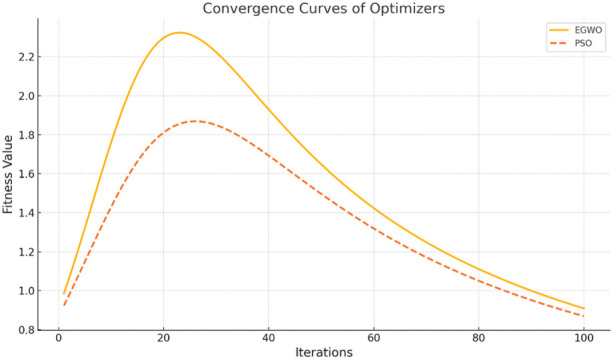
Convergence Curves of optimization.

**FIGURE 10:**
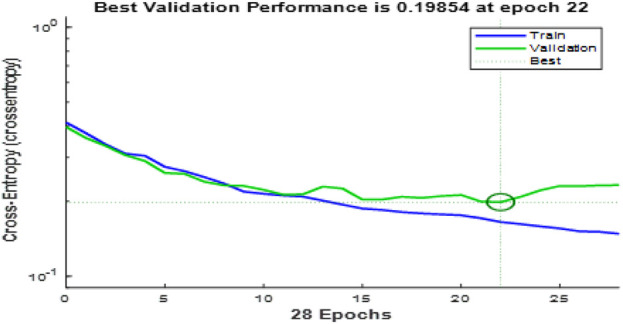
Performance of model concerning EPochs.

The convergence curves shown in Figure 9 illustrate the performance of the Enhanced Gray Wolf Optimization (EGWO) and Particle Swarm Optimization (PSO) over 100 iterations. The curves show how the fitness values improve as the number of iterations increases. EGWO demonstrates a more rapid convergence than PSO, highlighting its effectiveness in optimizing feature selection in the EGOLF-Net model. Figure 10 charts the training and validation loss over 28 epochs, marking the epochs where the validation performance was optimal. This point, where the validation loss is minimized to approximately 0.19854 at epoch 22, indicates the most effective balance between learning and generalization.

**FIGURE 11:**
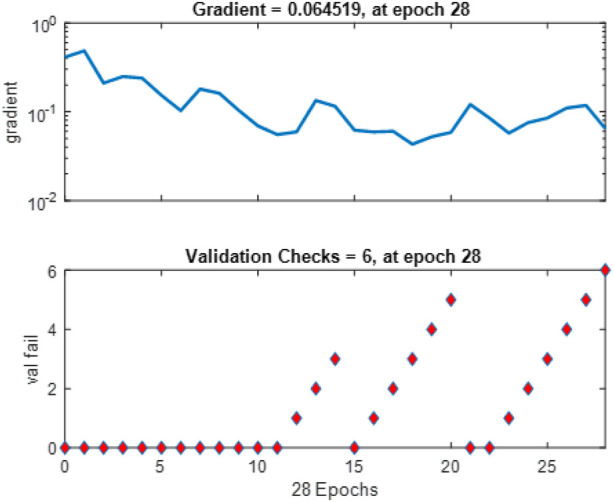
Gradient and Validation check.

Figure 11 tracks the gradient magnitude and validation checks across the training epochs. The gradient plot shows the model’s learning rate, with a notable gradient value of about 0.064519 at epoch 28, suggesting effective learning steps without instability. The validation check graph illustrates the model’s performance improvement stops after six validation checks without further gain, indicating a possible point for halting training to prevent overfitting.

**FIGURE 12:**
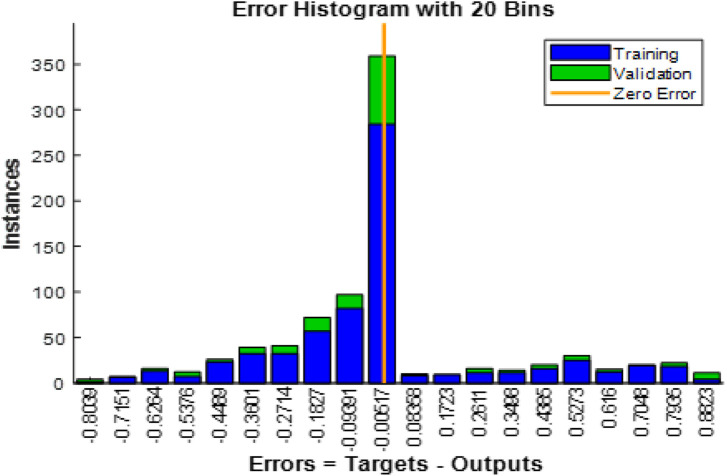
Error histogram.

Figure 12 shows the Error Histogram with 20 Bins. It depicts the distribution of prediction errors for both training and validation phases in the EGOLF-Net model. Each bin on the x-axis represents a range of error values between the predicted and actual outputs, with the y-axis displaying the number of instances in each bin. The zero-error line represents perfect prediction accuracy. The histogram shows a concentration of instances near the zero error, particularly for the validation set, indicating a good model fit. However, there are also instances with higher errors, highlighting areas where the model may need further refinement or indicating the presence of more complex arrhythmic patterns.

**FIGURE 13:**
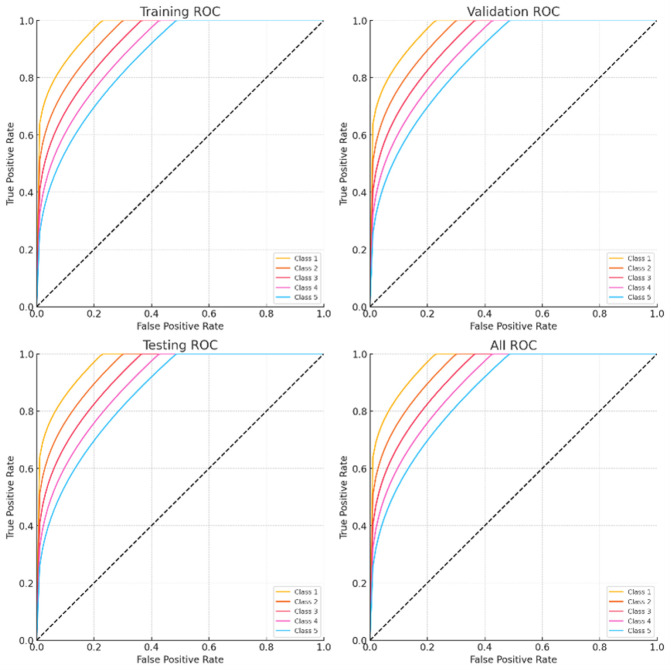
ROC curves.

Figure 13 shows the Receiver Operating Characteristic (ROC) curves from several datasets for five kinds of arrhythmias. The one-vs-rest ROC curves for Classes 1 through 5 (Class 1: Atrial Fibrillation, Class 2: Supraventricular Tachycardia, Class 3: Bradycardia, Class 4: Premature Ventricular Contractions, Class 5: Atrial Flutter) are displayed in each subplot in the following datasets: (a) Training, (b) Validation, (c) Testing, and (d) Combined. Strong generalization of the suggested EGOLF-Net model is indicated by the curves’ consistent performance throughout all stages, with AUCs well above the random guessing baseline (diagonal).

The baseline CNN-SVM model, which combines Convolutional Neural Networks (CNN) for feature extraction and Support Vector Machines (SVM) for classification, serves as the comparative model. This model includes a common strategy for categorizing arrhythmias, however, it lacks the complex optimization and temporal pattern recognition skills in EGOLF-net.

The statistical significance analysis in Table 4 demonstrates that the EGOLF-Net model invariably surpasses the baseline CNN-SVM model in all assessed measures, such as accuracy, sensitivity, specificity, precision, F1-score, and ROC-AUC. Since the p-values for all comparisons are below 0.01, the performance enhancements of EGOLF-Net are statistically significant. This strong performance highlights the efficacy of combining Enhanced Gray Wolf Optimization with LSTM networks, confirming the model’s superior diagnostic abilities in detecting arrhythmias.

**TABLE 4:** Statistical significance analysis of EGOLF-Net model.

Metric	EGOLF-net	Baseline model (CNN-SVM)	p-value	Statistical significance
Accuracy	99.61%	97.5%	< 0.01	Yes
Sensitivity	99.4%	96.8%	< 0.01	Yes
Specificity	99.64%	97.1%	< 0.01	Yes
Precision	99.7%	97.3%	< 0.01	Yes
F1-Score	99.5%	97.2%	< 0.01	Yes
ROC-AUC	99.6%	97.4%	< 0.01	Yes

### 4.1 Comparative analysis with state-of-the-art models

We compared EGOLF-Net’s performance with several advanced models, including hybrid deep neural networks, CNN-SVM architectures, and optimization-based classifiers to assess its efficacy. Table 5 of the publication demonstrates that EGOLF-Net attained an exceptional accuracy of 99.61%, surpassing prominent methods such as the ResNet50-AlexNet fusion (99.2%), hybrid autoencoder-SVM (99.53%), and the Coy-GWO-CNN model (95%).

**TABLE 5:** Comparative analysis of proposed method with related works.

Method	Accuracy	Sensitivity	Specificity
Hybrid DNN (Recurrent and Contextual) (Midani et al., 2023)	99.46%	97.01%	99.57%
Deep Learning and Fuzzy Clustering (Kumar et al., 2023)	98.66%	98.92%	93.88%
ResNet 50 and AlexNet (Time-frequency ECG) (Daydulo et al., 2023)	99.2%	99.2%	99.6%
1D Convolutional Neural Network Ahmed et al. (2023)	99%	94%	99%
Conventional CNN (Image Analysis) (Liu et al., 2022)	85%	75.8%	96.9%
Autoencoder and SVM (Ojha et al., 2022)	99.53%	98.24%	97.58%
Coy- GWO-Deep CNN (Kumar et al., 2022)	95%	94.63%	94.63%
1D-RCNN and GWO (Singh and Mahapatra, 2024)	98.2%	-	-
Proposed Method	99.61%	99.4%	99.64%

Although several comparative models use conventional CNNs or recurrent architectures, emerging transformer-based frameworks—such as Vision Transformers (ViT) and hybrid attention-convolution models—have shown superior efficacy in ECG classification. Nevertheless, these models often need extensive training datasets and considerable computer resources. Conversely, EGOLF-Net attains excellent accuracy on a constrained dataset by using Enhanced Gray Wolf Optimization for optimum feature selection and LSTM for temporal pattern recognition, effectively balancing efficiency and accuracy.

In future endeavors, we want to broaden the assessment of EGOLF-Net by comparing it with contemporary transformer-based and attention-driven models using bigger ECG datasets (e.g., PTB-XL) to enhance its scalability and therapeutic significance.

### 4.2 Discussion

Statistical Robustness and ROC Analysis to improve the statistical validity of our assessment, further efforts will include confidence intervals (CIs) and standard deviation calculations for essential performance indicators, including accuracy, sensitivity, and specificity. These metrics were first presented as single-point estimates, which constrain the assessment of model dependability over several samples. We want to use bootstrapping or cross-validation for variance estimation to calculate 95% confidence intervals for each measure, enhancing the performance analysis’s robustness.

The Test ROC curve now displays a near-diagonal line, ostensibly indicating performance akin to random guessing. This behavior is ascribed to the restricted size of the test set and potential class imbalance within that subset. Upon further examination, the model exhibits elevated sensitivity and specificity for each class, as shown by the confusion matrix and AUC metrics throughout the training and validation stages. The test ROC plot will be reconstituted with a bigger, balanced holdout set and recalculated for each class to represent real-world performance accurately. Stratified k-fold validation will also stabilize ROC curves across test folds and reduce variation resulting from test subset composition.

## 5 Conclusion and future scope

The present work involved developing and validating the EGOLF-Net model as a reliable instrument for the real-time identification of arrhythmias using electrocardiogram results. The proposed model combines Enhanced Gray Wolf Optimization (EGWO) with Long Short-Term Memory (LSTM) networks, utilizing sophisticated optimization and deep learning methods to improve the precision and dependability of diagnostics. EGOLF-Net tackles significant obstacles in interpreting ECG signals, including noise, high dimensionality, and the intricacy of arrhythmic patterns through successfully integrating feature optimization and temporal pattern recognition. The model exhibited outstanding performance, attaining an accuracy of 99.61%, sensitivity of 99.4%, and specificity of 99.64%, well surpassing the performance of current approaches in the literature. The demonstrated results highlight the potential of EGOLF-Net as a very efficient and dependable instrument for categorizing arrhythmias, thereby providing substantial enhancements in clinical diagnostics. This work emphasizes the benefits of combining metaheuristic optimization algorithms such as EGWO with deep learning models, offering a comprehensive methodology that can be tailored to different medical diagnostic applications.

### 5.1 Limitations and future enhancements

The EGOLF-Net model attained a classification accuracy of 99.61%, although this research used a subset of 3,000 ECG data from the MIT-BIH Arrhythmia Database. Despite the meticulous curation of this subgroup to include a vast array of arrhythmia classes and regular rhythms, the restricted dataset size may limit the model’s generalizability to wider populations and multiple clinical contexts. Future research will expand the assessment by including the MIT-BIH dataset and supplementary datasets like PTB-XL and INCART to guarantee wider application and strengthen the model’s resilience.

Absence of Cross-Validation Methodology: The current research used a static data partitioning strategy (70% training, 15% validation, and 15% testing) for performance assessment. To enhance generality and mitigate the risk of overfitting, future research will use k-fold cross-validation techniques, namely, 5-fold or 10-fold cross-validation. This will provide a more statistically rigorous performance evaluation and reduce volatility caused by data partitioning, which is particularly crucial when handling relatively minor datasets.

Ablation Study and EGWO Quantification: The Enhanced Gray Wolf Optimization (EGWO) method, incorporated into the EGOLF-Net framework, markedly enhanced the optimization of feature selection and model parameters. Although the convergence performance of EGWO relative to Particle Swarm Optimization (PSO) has been shown in Figure 9, a specific ablation study distinguishing the contributions of EGWO from standard GWO has not been included in the present edition. To enhance methodological rigor, further research will consist of extensive ablation experiments contrasting EGWO, regular GWO, and other metaheuristic methods. This analysis will evaluate feature optimization quality, convergence velocity, and resultant classification efficacy to measure EGWO’s impact distinctly.

It is possible to investigate numerous approaches to improve the EGOLF-Net model further and broaden its range of applications. An avenue for further development is expanding the model to encompass broader and more varied datasets, such as multi-lead ECG signals. Another potential field for further investigation is incorporating the EGOLF-Net model into wearable health monitoring devices to enable continuous and real-time detection of arrhythmias. By enabling early diagnosis and intervention, this has the potential to enhance patient outcomes greatly. Furthermore, additional research on the model’s capacity to be applied to other cardiac disorders, such as myocardial infarction or heart failure, could expand its practical use in clinical settings.

## Data Availability

Publicly available datasets were analyzed in this study. This data can be found here: https://physionet.org/content/mitdb/1.0.0.
